# Supplementary material for: Diagnostic Accuracy of High-Grade Intraepithelial Papillary Capillary Loops by Narrow Band Imaging for Early Detection of Oral Malignancy: A Cross-Sectional Clinicopathological Imaging Study
Source: Cancers (Basel). 2022 May 13;14(10):2415. doi: 10.3390/cancers14102415 (PMC9139655; doi:10.3390/cancers14102415)
Supplement: Supplementary file 1 [file cancers-14-02415-s001.zip › cancers-1708867-supplementary.pdf]

Supplemental Data

Participant Characteristics.

Participants	Gender	Age	Histopathological diagnosis by incisional biopsy	Site	Clinical appearance (homogeneous /non-homogeneous)	Type of intraoral epithelial papillary capillary loops (IPCL)
**1**	F	67	OLP	Oral vestibule	Homogeneous	I
**2**	F	67	OLP	Buccal mucosa	Homogeneous	II
**3**	F	78	OLP	Buccal mucosa	Non-Homogeneous	III
**4**	F	39	OLP	Buccal mucosa	Homogeneous	II
**5**	F	69	OLP	Buccal mucosa	Homogeneous	I
**6**	F	60	OLP	Buccal mucosa	Non-Homogeneous	II
**7**	F	79	OLP	Buccal mucosa	Non-Homogeneous	III
**8**	M	44	OLP	Buccal mucosa	Homogeneous	II
**9**	M	65	OLP	Buccal mucosa	Non-Homogeneous	III
**10**	F	76	OLP	Buccal mucosa	Homogeneous	II
**11**	M	53	OLP	Buccal mucosa	Non-Homogeneous	II
**12**	F	52	OLP	Tongue	Homogeneous	II
**13**	F	38	OLP	Buccal mucosa	Non-Homogeneous	II
**14**	F	35	OLP	Buccal mucosa	Non-Homogeneous	III
**15**	F	65	OLP	Buccal mucosa	Non-Homogeneous	I
**16**	F	63	OLP	Buccal mucosa	Non-Homogeneous	I

**17**	M	71	Oral epithelial hyperplasia	Tongue	Homogeneous	I
**18**	F	64	Proliferative verrucous leukoplakia	Hard palate	Non-Homogeneous	IV
**19**	F	74	Oral epithelial hyperplasia	Hard palate	Homogeneous	0
**20**	M	77	Oral epithelial dysplasia (mild)	Tongue	Homogeneous	0
**21**	F	79	Oral epithelial dysplasia (moderate)	Tongue	Homogeneous	0
**22**	F	38	Oral epithelial hyperplasia	Tongue	Homogeneous	I
**23**	F	79	Oral epithelial hyperplasia	Gingiva	Homogeneous	I
**24**	M	62	Oral epithelial hyperplasia	Buccal mucosa	Homogeneous	I
**25**	F	58	Oral epithelial hyperplasia	Tongue	Homogeneous	0
**26**	M	48	Oral epithelial hyperplasia	Hard palate	Homogeneous	0
**27**	F	52	Oral epithelial hyperplasia	Gingiva	Homogeneous	I
**28**	M	65	Oral epithelial	Buccal mucosa	Homogeneous	III

			hyperplasia			
**29**	F	67	Hyperkeratosis	Buccal mucosa	Non-Homogeneous	II
**30**	F	47	Oral epithelial hyperplasia	Gingiva	Homogeneous	0
**31**	F	60	Oral epithelial hyperplasia	Gingiva	Homogeneous	I
**32**	M	81	Oral epithelial dysplasia (mild~moderate)	Buccal mucosa	Homogeneous	0
**33**	F	61	Oral epithelial hyperplasia	Buccal mucosa	Homogeneous	II
**34**	M	55	Oral epithelial hyperplasia	Hard palate	Homogeneous	I
**35**	F	59	Oral epithelial hyperplasia	Gingiva	Homogeneous	I
**36**	M	47	Oral epithelial hyperplasia	Gingiva	Homogeneous	I
**37**	M	60	Oral epithelial hyperplasia	Gingiva	Homogeneous	0
**38**	F	65	Oral epithelial hyperplasia	Gingiva	Homogeneous	I
**39**	F	79	Oral epithelial hyperplasia	Buccal mucosa	Homogeneous	I

**40**	M	44	Oral epithelial hyperplasia	Gingiva	Homogeneous	I
**41**	M	73	Oral epithelial hyperplasia	Hard palate	Homogeneous	I
**42**	M	72	Oral epithelial hyperplasia	Gingiva	Homogeneous	0
**43**	M	69	Oral epithelial hyperplasia	Buccal mucosa	Homogeneous	I
**44**	M	47	Oral epithelial dysplasia (moderate)	Tongue	Non-Homogeneous	Ⅳ
**45**	M	65	Proliferative verrucous leukoplakia	Tongue	Non-Homogeneous	Ⅲ
**46**	M	70	Oral epithelial hyperplasia	Tongue	Homogeneous	Ⅱ
**47**	F	63	Oral epithelial dysplasia (moderate)	Tongue	Non-Homogeneous	Ⅲ
**48**	M	78	Carcinoma in situ	Oral floor	Homogeneous	Ⅲ
**49**	F	87	OSCC	Gingiva	Non-Homogeneous	Ⅲ
**50**	M	73	OSCC	Tongue	Non-Homogeneous	Ⅳ
**51**	F	48	OSCC	Tongue	Non-Homogeneous	Ⅲ
**52**	M	74	OSCC	Tongue	Non-Homogeneous	Ⅳ
**53**	M	81	OSCC	Tongue	Non-Homogeneous	Ⅳ
**54**	F	83	OSCC	Hard palate	Non-Homogeneous	Ⅲ

**55**	F	79	OSCC	Tongue	Non-Homogeneous	III
**56**	M	34	OSCC	Tongue	Non-Homogeneous	IV
**57**	M	57	OSCC	Tongue	Non-Homogeneous	IV
**58**	F	50	OSCC	Tongue	Non-Homogeneous	IV
**59**	M	72	OSCC	Tongue	Non-Homogeneous	IV
**60**	M	47	OSCC	Gingiva	Non-Homogeneous	IV
